# Clinical experience of craniospinal intensity-modulated spot-scanning proton therapy using large fields for central nervous system medulloblastomas and germ cell tumors in children, adolescents, and young adults

**DOI:** 10.1093/jrr/rrz022

**Published:** 2019-05-21

**Authors:** Takayuki Hashimoto, Shinichi Shimizu, Seishin Takao, Shunsuke Terasaka, Akihiro Iguchi, Hiroyuki Kobayashi, Takashi Mori, Takaaki Yoshimura, Yuto Matsuo, Masaya Tamura, Taeko Matsuura, Yoichi M Ito, Rikiya Onimaru, Hiroki Shirato

**Affiliations:** 1 Department of Radiation Medicine, Faculty of Medicine, Hokkaido University; 2 Global Station for Quantum Medical Science and Engineering, Global Institution for Collaborative Research and Education (GI-CoRE), Hokkaido University; 3 Department of Radiation Oncology, Faculty of Medicine, Hokkaido University; 4 Proton Beam Therapy Center, Hokkaido University Hospital; 5 Department of Neurosurgery, Faculty of Medicine, Hokkaido University; 6 Department of Pediatrics, Faculty of Medicine, Hokkaido University; 7 Department of Radiation Oncology, Hokkaido University Hospital; 8 Division of Quantum Science and Engineering, Faculty of Engineering, Hokkaido University; 9 Department of Statistical Data Science, The Institute of Statistical Mathematics

**Keywords:** craniospinal irradiation, intensity-modulated spot-scanning proton therapy, adolescents and young adults, vertebral body sparing, hematologic toxicity, beam delivery time

## Abstract

The outcomes of intensity-modulated proton craniospinal irradiation (ipCSI) are unclear. We evaluated the clinical benefit of our newly developed ipCSI system that incorporates two gantry-mounted orthogonal online X-ray imagers with a robotic six-degrees-of-freedom patient table. Nine patients (7–19 years old) were treated with ipCSI. The prescribed dose for CSI ranged from 23.4 to 36.0 Gy (relative biological effectiveness) in 13–20 fractions. Four adolescent and young adult (AYA) patients (15 years or older) were treated with vertebral-body-sparing ipCSI (VBSipCSI). Myelosuppression following VBSipCSI was compared with that of eight AYA patients treated with photon CSI at the same institution previously. The mean homogeneity index (HI) in the nine patients was 0.056 (95% confidence interval: 0.044–0.068). The mean time from the start to the end of all beam delivery was 37 min 39 s ± 2 min 24 s (minimum to maximum: 22 min 49 s – 42 min 51 s). The nadir white blood cell, hemoglobin, and platelet levels during the 4 weeks following the end of the CSI were significantly higher in the VBSipCSI group than in the photon CSI group (*P* = 0.0071, 0.0453, 0.0024, respectively). The levels at 4 weeks after the end of CSI were significantly higher in the VBSipCSI group than in the photon CSI group (*P* = 0.0023, 0.0414, 0.0061). Image-guided ipCSI was deliverable in a reasonable time with sufficient HI. Using VBSipCSI, AYA patients experienced a lower incidence of serious acute hematological toxicity than AYA patients treated with photon CSI.

## INTRODUCTION

Craniospinal irradiation (CSI) plays an essential role in the management of central nervous system malignancies such as medulloblastomas and germ cell tumors, which have the propensity to disseminate throughout the neuroaxis. It has been documented that proton beam therapy (PBT) is superior to photon beam therapy in reducing the dose to normal tissues in CSI [1, 2]. From a biological point of view, intensity-modulated proton craniospinal irradiation (ipCSI) is expected to be superior to passive scattering (PS)-PBT for children, and adolescent and young adult (AYA) patients, because it leads to less frequent contamination of neutrons [3]. Recently, ipCSI has been shown to be superior to CSI using PS-PBT (psCSI) to improve the dose distribution, especially at the junction of the fields [4, 5]. However, the robustness of ipCSI can deteriorate if the treatment delivery time of ipCSI is longer than that for psCSI. We have developed an ipCSI system that incorporates two gantry-mounted orthogonal online x-ray imagers with a robotic six-degrees-of-freedom patient table. In this study, we evaluated the time required to perform ipCSI using the system for all the patients who required CSI during the study period.

Moreover, in our literature search we did not find any research regarding the clinical results of ipCSI. Therefore, in this study we investigated the clinical results of child and AYA patients who were treated with ipCSI. According to several dosimetric and clinical studies, vertebral-body-sparing (VBS) psCSI was reported to be associated with fewer acute hematologic adverse events compared with photon CSI [2, 6–8]. Recently Giantsoudi *et al.* showed that ipCSI has a dosimetric advantage over psCSI in terms of VBS [9]. However, studies demonstrating the clinical results of VBS using ipCSI (VBSipCSI) are currently lacking in the literature. We adapted VBS using ipCSI for AYA patients, and we compared the hematological adverse reactions experienced by these AYA patients with those of AYA patients treated with photon CSI previously in our institution.

## MATERIALS AND METHODS

### Study design

We conducted a retrospective study of patients who were treated with ipCSI at Hokkaido University Hospital between July 2016 and January 2018. We compared the hematological toxicity experienced by the AYA patients in this period with that of AYA patients who were treated with photon CSI at the same institution between November 2004 and October 2015.

### Patients

Nine patients with histologically confirmed medulloblastomas (*n* = 6) and germ cell tumors (GCTs) (*n* = 3), treated with ipCSI at Hokkaido University Hospital from July 2016 to January 2018, were retrospectively analyzed. The characteristics of the patients are listed in Table 1. The median age at enrollment was 11 years (7–19 years). For four AYA cases, VBS was used in whole-spinal irradiation. One of the AYA patients had previously received whole-brain irradiation 6 years prior, and therefore the patient did not receive whole-brain irradiation during this period. For five patients under 15 years of age, VBS was not used.

**Table 1.** Characteristics and treatment parameters of patients who received ipCSI

Case no.	Age (year)	Sex	Histology	Chemotherapy regimen			Total PBT dose [Gy (RBE)]			Acute hematological AE within 4 weeks after CSI		
				Pre CSI	Concurrent CSI	Post CSI	CSI	Intracranial boost	Spinal boost	WBC	Hb	Platelet
AYA cases												
1	19	M	GCT	(IFOS-CDDP-VP-16) × 6	none	none	^a^23.4	^b^none	21.6	Gr. 2	Gr. 2	Gr. 0
2	16	M	GCT	(IFOS-CDDP-VP-16) × 1	none	(IFOS-CDDP-VP-16) × 5	30.6	30.6	none	Gr. 0	Gr. 1	Gr. 0
3	17	F	GCT	(IFOS-CDDP-VP-16) × 1	none	(IFOS-CDDP-VP-16) × 5	30.6	30.6	none	Gr. 0	Gr. 0	Gr. 0
4	17	M	MB	none	(IFOS-CDDP-VP-16) × 1	(IFOS-CDDP-VP-16) × 5	23.4	32.4	none	Gr. 3	Gr. 1	Gr. 1
Patients < 15 years of age												
5	9	F	MB	none	(CBDCA-VCR) × 1	(CPM-VCR) × 6	36.0	19.8	none	Gr. 3	Gr. 3	Gr. 2
6	10	M	MB	none	none	n/a	23.4	30.6	none	Gr. 2	Gr. 1	Gr. 0
7	9	M	MB	(VCR-CDDP-VP-16-CPM) × 3(CBDCA-Thio + PBSCR) × 3	none	n/a	36.0	18.0	9.0	Gr. 2	Gr. 1	Gr. 1
8	7	F	MB	none	none	n/a	23.4	30.6	none	Gr. 2	Gr. 0	Gr. 1
9	11	M	MB	none	VCR × 1	(CDDP-VCR-CPM) × 8	23.4	32.4	none	Gr. 3	Gr. 2	Gr. 0

^a^Only whole-spinal irradiation conducted. ^b^Whole-cranial irradiation of 25.2 Gy in 14 fractions and boost irradiation of 19.8 Gy in 11 fractions using photon beams 6 years before PBT. PBT = proton beam therapy, RBE = relative biological effectiveness, CSI = craniospinal irradiation, ipCSI = intensity-modulated proton craniospinal irradiation, AE = adverse events, M = male, F = female, WBC = white blood cell, Hb = hemoglobin, AYA = adolescent and young adult, Gr. = Grade, GCT = germ cell tumor, MB = medulloblastoma, IFOS = ifosfamide, CDDP = cisplatin, VP-16 = etoposide, VCR = vincristine, CPM = cyclophosphamide, CBDCA = carboplatin, Thio = thio-TEPA, PBSCR = peripheral blood stem cell rescue.

From November 2004 to October 2015, there were eight AYA patients treated with photon CSI for whom hematologic acute toxicity data was available for the period of 4 weeks after the end of CSI. Patient backgrounds and treatment parameters for photon CSI are listed in Table 2.

**Table 2.** Characteristics, treatment parameters, and acute hematological adverse events of AYA patients who received photon CSI

Case no.	Age (years)	Sex	Histology	Chemotherapy regimen			Total dose (Gy)			Acute hematological AEs within 4 weeks after CSI		
				Pre CSI	Concurrent CSI	Post CSI	CSI	Intracranial boost	Spinal boost	WBC	Hb	Platelet
1	25	M	GCT	(IFOS-CDDP-VP-16) × 3	none	none	25.2	25.2	none	Gr. 2	Gr. 2	Gr. 1
2	26	F	PNET	(IFOS-CDDP-VP-16) × 1	none	(IFOS-CDDP-VP-16) × 4	25.2	28.8	none	Gr. 2	Gr. 2	Gr. 3
3	28	M	GCT	(CBDCA+VP-16) × 2	none	none	25.2	25.2	none	Gr. 3	Gr. 2	Gr. 1
				(IFOS-CDDP-VP-16) × 2								
4	26	F	PNET	(CBDCA+VP-16) × 1	none	none	36.0	none	none	Gr. 2	Gr. 3	Gr. 2
5	21	M	GCT	(CBDCA+VP-16) × 3	none	none	23.4	27.0	none	Gr. 3	Gr. 1	Gr. 1
6	18	F	MB	none	(IFOS-CDDP-VP-16) × 1	(IFOS-CDDP-VP-16) × 4	23.4	32.4	none	Gr. 3	Gr. 2	Gr. 0
7	28	F	PNET	none	none	(CDDP+VP-16) × 2	25.2	28.8	none	Gr. 2	Gr. 1	Gr. 0
						TMZ × 2						
8	24	M	GCT	(IFOS-CDDP-VP-16) × 4	none	none	25.2	none	none	Gr. 3	Gr. 1	Gr. 0

AYA = adolescent and young adult, CSI = craniospinal irradiation, ipCSI = intensity-modulated proton craniospinal irradiation, AEs = adverse events, M = male, F = female, WBC = white blood cell, Hb = hemoglobin, GCT = germ cell tumor, PNET = primitive neuroectodermal tumor, MB = medulloblastoma, IFOS = ifosfamide, CDDP = cisplatin, VP-16 = etoposide, CBDCA = carboplatin, TMZ = temozolomide.

The present study was reviewed and approved by the institutional review board of Hokkaido University Hospital. Written informed consent, which included general consent, was obtained from the patient or a person with parental authority before the treatment.

### Proton beam therapy

**Figure 1.**
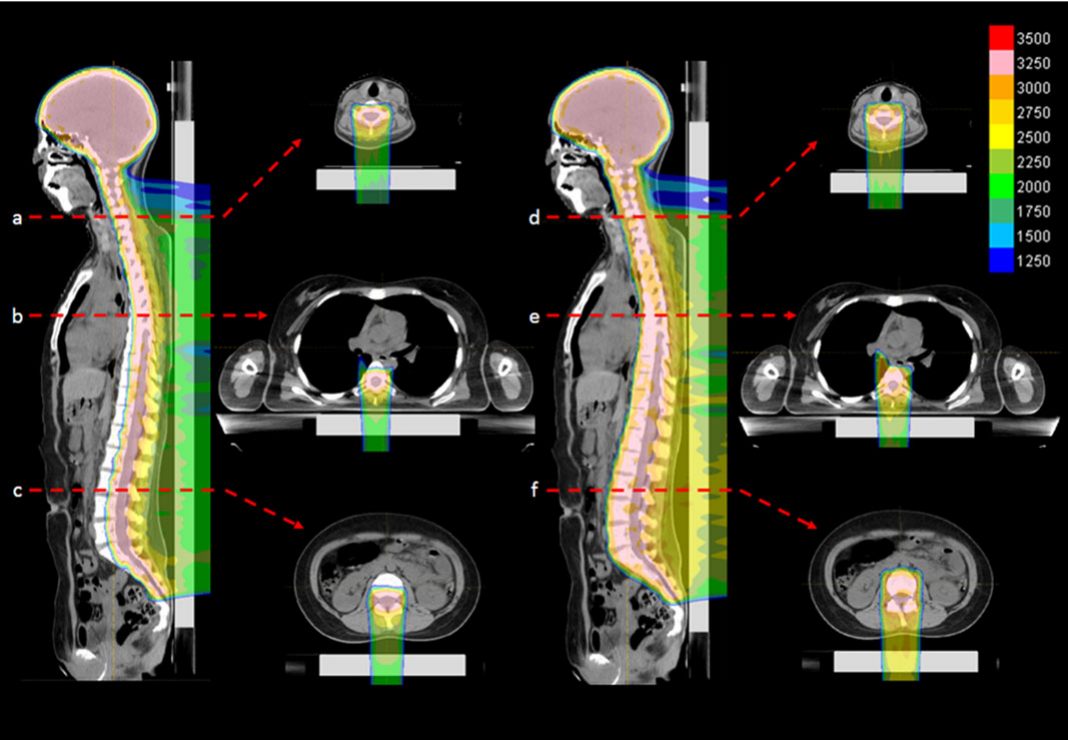
(Left) An example of actual dose distribution of VBSipCSI in an adolescent patient (Case no. 3 in Table 1): cervical spine (top), thoracic spine (middle) and lumbar spine (bottom) levels. (Right) Simulated dose distribution of ipCSI without VBS for the same patient, which was not used for the patient.

PROBEAT-RT system (Hitachi, Ltd, Tokyo, Japan), a synchrotron-based proton therapy system dedicated to discrete spot-scanning techniques [10–12], was developed and used in this study. The maximal field size was 30 × 40 cm^2^ at the isocenter. Two laterally opposed fields were used for whole-brain irradiation. One or two posterior fields were used for whole-spine irradiation. The intensity-modulation technique was used in whole-spine irradiation for all patients. For VBSipCSI, posterior–anterior beams were designed to spare the anterior part of the vertebral body (Fig. 1). The relative biological effectiveness (RBE) was assumed to be 1.1 throughout the spread-out peak, although we noticed that the RBE at the distal edge of the spread-out peak was higher than that [13]. The effect of the higher RBE was regarded as negligible for this analysis, because the absorbed dose at the distal edge was much lower than the prescribed dose at the spread-out peak.

A clinical target volume 1 (CTV1), which included the whole intracranial space and the entire thecal sac, was contoured. The prescribed dose of 95% or 99% of the CTV1 for the whole craniospinal region was 23.4 Gy (RBE) for the average-risk medulloblastoma, 30.6 Gy (RBE) for the poor-prognosis germ cell tumors, 36.0 Gy (RBE) for the high-risk medulloblastoma, and 54.0–61.2 Gy (RBE) for the primary site using 1.8 Gy (RBE) per fraction. Boost irradiation to a part of the spinal region was performed in two patients with a total dose of 45.0 Gy (RBE). A clinical target volume 2 (CTV2), which included the total vertebral body and CTV1, was also contoured. CTV2 was used in the treatment planning for ipCSI without VBS in patients under 15 years of age. A 3D treatment planning system (TPS), VQA (Hitachi, Ltd, Tokyo, Japan) was used for treatment planning. For both VBSipCSI and ipCSI without VBS, the craniocaudal margin of the beam-specific planning target volume (bPTV) for CTV1 was 5.0 mm. The lateral margin of the bPTV for CTV1 was 3.0 mm and 7.0 mm for whole-brain and spinal irradiation, respectively. The distal margins of the bPTV for the CTV1 along the beam direction were 3.5% of the ranges to the distal/proximal surfaces of the CTV1 plus 1 mm for VBSipCSI. As a result, the anterior part of the vertebral body could be spared with VBSipCSI.

For ipCSI without VBS, the distal margins of the bPTV for the CTV2 along the beam direction were determined to include the CTV2, with 80% of the prescribed dose at the distal fall-off of the spread-out peak. Dose distribution in the CTV1 was planned to be as homogeneous as possible by using an optimization algorithm, especially at the junction of the fields. An optimization algorithm for overlapping-field plans, which had been developed by Inaniwa *et al.* [14], was used for suppressing the dose gradient within the CTV in order to prevent a steep dose gradient and to mitigate hot and cold spots at the field junction.

Regarding the set-up of the patient, bone-matching between digitally reconstructed images and two gantry-mounted orthogonal online X-ray imagers with a robotic six-degrees-of-freedom patient table is used for quick online adjustments. The bone-matching was utilized in the set-up for all portals, including tandem portals for spinal irradiation in all patients.

### Photon therapy

During the period between 2004 and 2015, 6 or 10 MV photon beams delivered by a Varian Clinac 2300, iX linear accelerator (Varian Medical Systems, Inc., Palo Alto, CA), and Mitsubishi EXL (Mitsubishi Electric Corporation, Tokyo, Japan) were used for CSI. Treatment characteristics are shown in Table 2. The dose for whole-spinal irradiation was prescribed at the mean depth of the spinal cord. Conformal 3D radiotherapy was administered according to the planning target volume (PTV), which consisted of the CTV2 with the addition of a 5.0 mm margin.

### Chemotherapy

The chemotherapy regimen and schedules used with ipCSI and those used with photon CSI are shown in Table 1 and Table 2, respectively. For AYA patients, the ICE regimen (consisting of ifosfamide, cisplatin and etoposide) [15] was used in the ipCSI groups. Patients under 15 years of age received a different regimen. In the photon CSI group, the ICE regimen, or carboplatin and etoposide was used.

### Data collection and assessment

Dose–volume statistics based on the histogram were evaluated in all cases. Dose–volume statistics of the CTV1, vertebra, heart, lungs, and abdominal cavity were assessed. The conformity index was defined as the volume that was covered by the reference isodose divided by the CTV1. The homogeneity index (HI) was calculated using the formula HI = (D2 – D98)/D50, where D2, D98 and D50 were the minimum doses delivered to 2%, 98% and 50% of the CTV1. We delineated from the first cervical vertebral body to the lumbar vertebral bodies, and dose–volume histograms (DVHs) were used to evaluate the dosimetric parameters.

JMP software (version 12, SAS Institute Inc., Cary, NC) was used for the statistical analysis. Mean doses administered to the vertebral bodies were compared between VBSipCSI and ipCSI without VBS in the same AYA patient groups using paired *t*-tests.

The beam delivery time, the time from the start to the end of all beam delivery, and the time spent in the treatment room for ipCSI were calculated from all log data in the PBT system and the clocks in the treatment rooms for all patients except one.

Severity of acute hematological toxicity was graded using the Common Terminology Criteria for Adverse Events (CTCAE) version 4.0. The white blood cell (WBC) count, hemoglobin level, and platelet counts were measured at the start of, during, and at 4 weeks after the end of CSI. These were measured two times a week in principle. The nadir of these values during the 4 weeks and the values at the 4 weeks after the end of CSI were compared between the VBSipCSI and photon CSI groups. The differences in the mean blood counts between these groups were compared using the one-tailed *t*-test.

## RESULTS

Dose–volume statistics for all AYA patients are shown in Table 3 and for patients under 15 years of age in Table 4. Excellent dose homogeneity was obtained for all patients; the mean HI in the nine patients was 0.056 [95% confidence interval (CI): 0.044–0.068]. The irradiated volume percentages of the heart, lungs, and abdominal cavity were quite small in all nine patients.

**Table 3.** Dose–volume statistics of VBSipCSI, which were actually used, and those of ipCSI without VBS, which were calculated for simulation but not used, in the same four AYA patients

		AYA patients VBSipCSI Mean (95% CI)	AYA patients ipCSI without VBS Mean (95% CI)	*P*-value
^∗^CTV for CSI	Homogeneity index	0.047 (0.024–0.070)	0.055 (0.031–0.079)	*P* = 0.116
	Conformity index	1.776 (0.789–2.763)	2.058 (0.429–3.686)	*P* = 0.135
Total vertebral bodies	Mean dose, Gy (RBE)	14.7 (10.5–18.9)	26.7 (19.7–33.8)	*P* = 0.001
Cervical region	Mean dose, Gy (RBE)	23.1 (17.2–29.0)	26.4 (18.0–34.7)	*P* = 0.012
Thoracic region	Mean dose, Gy (RBE)	16.3 (11.4–21.2)	26.9 (20.1–33.6)	*P* = 0.001
Lumbar region	Mean dose, Gy (RBE)	11.3 (7.4–15.2)	26.7 (19.4–33.9)	*P* = 0.002
Heart	Mean dose, Gy (RBE)	0.0 (0.0–0.0)	0.8 (0.1–1.5)	*P* = 0.019
	V20 (%)	0.0 (0.0–0.0)	0.3 (−0.1–0.7)	*P* = 0.058
	V10 (%)	0.0 (0.0–0.0)	2.2 (0.2–4.2)	*P* = 0.020
	V5 (%)	0.0 (0.0–0.0)	5.0 (0.6–9.3)	*P* = 0.018
Lung	Mean dose, Gy (RBE)	0.8 (0.3–1.4)	1.6 (0.3–2.8)	*P* = 0.092
	V20 (%)	0.4 (−0.3–1.0)	2.7 (−1.0–6.4)	*P* = 0.053
	V10 (%)	2.8 (0.4–5.2)	5.8 (1.3–10.3)	*P* = 0.072
	V5 (%)	5.7 (1.9–9.5)	8.5 (2.9–14.1)	*P* = 0.156
Abdominal cavity	Mean dose, Gy (RBE)	0.1 (0.0–0.2)	0.5 (0.1–0.8)	*P* = 0.011
	V20 (%)	0.1 (0.0–0.2)	0.4 (−0.1–0.8)	*P* = 0.097
	V10 (%)	0.5 (0.3–0.6)	1.4 (0.1–2.7)	*P* = 0.047
	V5 (%)	1.0 (0.6–1.4)	2.6 (0.7–4.4)	*P* = 0.024

^∗^Whole brain and whole thecal sac, except one patient of whom CTV was only whole thecal sac. VBSipCSI = vertebral-body-sparing intensity-modulated proton craniospinal irradiation, AYA = adolescent and young adult, CI = confidence interval, CTV = clinical target volume, RBE = relative biological effectiveness.

**Table 4.** Dose–volume statistics of CSI for patients under 15 years of age

		Patients <15 years of age ipCSI without VBS, mean (95% CI)
CTV for CSI	Homogeneity index	0.063 (0.047–0.079)
	Conformity index	1.194 (1.058–1.330)
Heart	Mean dose, Gy (RBE)	0.5 (−0.2–1.3)
	V20 (%)	0.1 (−0.2–0.5)
	V10 (%)	1.2 (−1.5–3.9)
	V5 (%)	2.9 (−2.4–8.3)
Lung	Mean dose, Gy (RBE)	2.7 (1.7–3.8)
	V20 (%)	4.3 (0.6–7.9)
	V10 (%)	10.3 (7.1–13.5)
	V5 (%)	14.9 (11.3–18.4)
Abdominal cavity	Mean dose, Gy (RBE)	0.6 (0.2–1.0)
	V20 (%)	0.4 (−0.2–1.0)
	V10 (%)	2.0 (0.3–3.7)
	V5 (%)	3.8 (1.1–6.5)

CSI = craniospinal irradiation, ipCSI = intensity-modulated proton craniospinal irradiation, VBS = vertebral body sparing, CI = confidence interval, CTV = clinical target volume, RBE = relative biological effectiveness.

**Figure 2.**
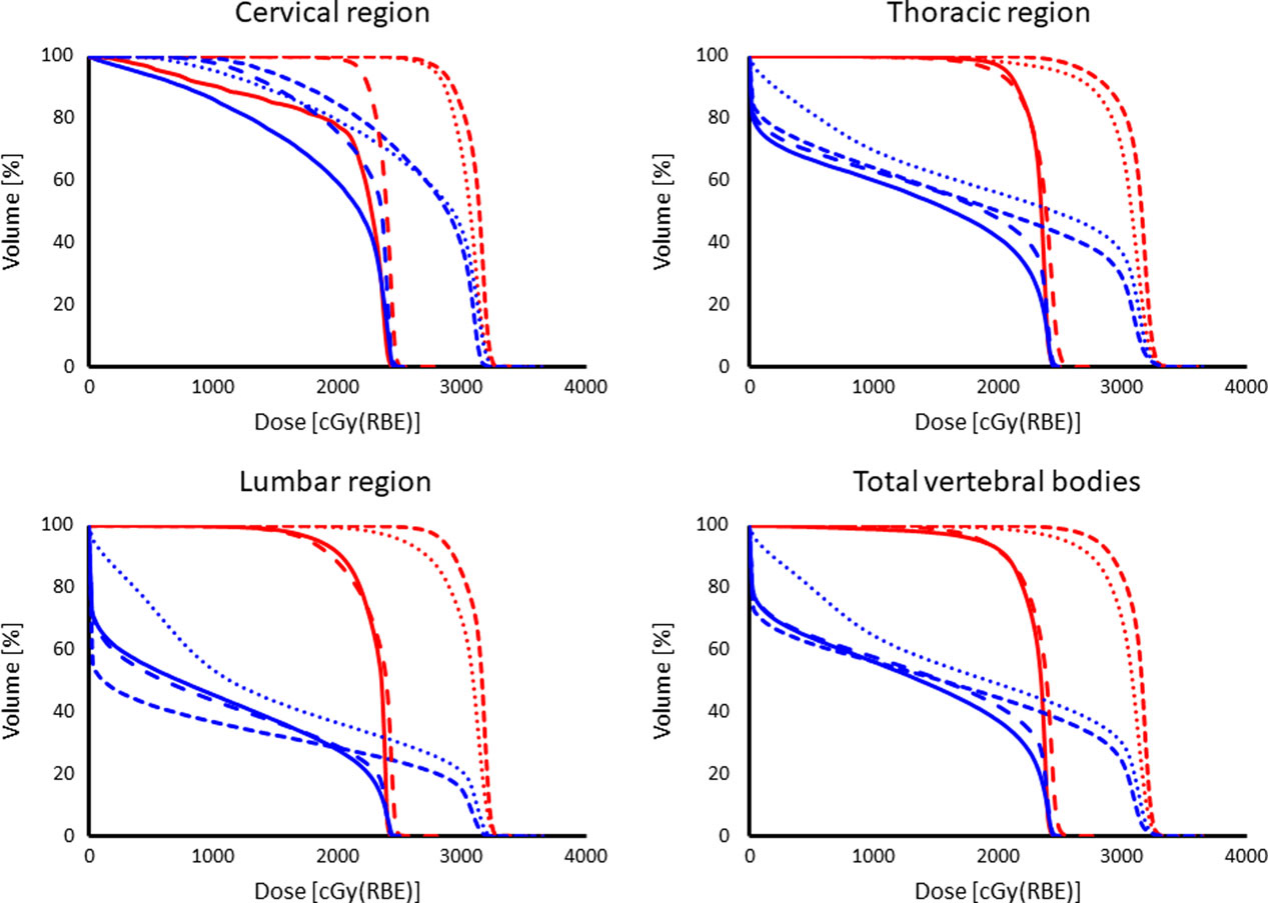
(blue) Actual dose-volume histograms (DVHs) of the vertebral bodies for four AYA patients (case No. 1–4) treated with VBSipCSI. Upper-left; cervical region, upper-right; thoracic region, lower-left; lumbar region, lower-right; total vertebral bodies. (red) DVHs of the simulated plans without VBS which were not used for the patient.

Statistical comparisons between VBSipCSI and ipCSI without VBS in the same AYA patient groups are shown in Table 3. There was no significant difference in the HI of the CTV between the VBSipCSI and ipCSI without VBS. For the AYA patients, DVHs for the vertebral bodies in the cervical, thoracic and lumbar regions, and the summation of these vertebral bodies (total vertebral bodies), which includes the area from the first cervical vertebral bone to the last lumbar vertebral bone are shown in Fig. 2. The mean ± one standard error (1SE) of the mean dose administered to the total vertebral bodies in AYA patients treated with VBSipCSI was 14.7 ± 1.3 Gy (RBE). This is significantly lower than the simulated dose based on the assumption that VBS had not been used for the same AYA patients (26.7 ± 2.2 Gy (RBE), *P* = 0.001). The dose was significantly lower with VBS in the cervical (*P* = 0.012), thoracic (*P* = 0.001) and lumbar (*P* = 0.002) regions, respectively. There was a significant reduction in the mean dose to the heart and abdominal cavity using VBS, although the dose to these organs at risk was quite small, even without VBS.

The time study was available for each patient except for one case (Case no. 1), whose log data was not available at the time of analysis (Table 5). The mean beam delivery time (min:s) was 7:32 (minimum to maximum: 6:43–8:39) for whole-brain irradiation and 4:54 (minimum to maximum: 3:20–5:43) for whole-spine irradiation.

**Table 5.** Time required for beam delivery, time from the start to the end of all beam delivery, and the time spent in the treatment room

Case no.	Time of the beam delivery to whole brain			Time of the beam delivery to whole spine			Time spent in the treatment room			Time from the start to the end of the whole beam delivery		
	Mean	Max	Min	Mean	Max	Min	Mean	Max	Min	Mean	Max	Min
AYA cases												
1	n/a	n/a	n/a	n/a	n/a	n/a	n/a	n/a	n/a	n/a	n/a	n/a
2	7 min 40 s	8 min 48 s	7 min 28 s	4 min 59 s	5 min 10 s	4 min 51 s	54 min 34 s	74 min 11 s	43 min 51 s	38 min 29 s	46 min 52 s	30 min 04 s
3	7 min 39 s	9 min 35 s	7 min 14 s	5 min 24 s	5 min 50 s	5 min 08 s	54 min 51 s	99 min 32 s	38 min 46 s	40 min 07 s	77 min 56 s	29 min 30 s
4	8 min 39 s	10 min 10 s	8 min 14 s	5 min 34 s	8 min 16 s	5 min 02 s	60 min 52 s	122 min 17 s	44 min 35 s	42 min 00 s	89 min 06 s	33 min 23 s
Mean	7 min 59 s	9 min 31 s	7 min 39 s	5 min 19 s	6 min 25 s	5 min 00 s	56 min 46 s	98 min 40 s	42 min 24 s	40 min 12 s	71 min 18 s	30 min 59 s
Under 15 years of age												
without general anesthesia												
5	6 min 43 s	7 min 00 s	6 min 35 s	3 min 20 s	4 min 16 s	3 min 13 s	43 min 24 s	82 min 02 s	33 min 01 s	22 min 49 s	34 min 28 s	18 min 39 s
6	7 min 10 s	7 min 54 s	6 min 56 s	5 min 43 s	6 min 23 s	5 min 29 s	60 min 11 s	86 min 12 s	41 min 36 s	42 min 34 s	57 min 33 s	33 min 09 s
7	7 min 27 s	8 min 16 s	7 min 14 s	5 min 13 s	5 min 37 s	5 min 04 s	56 min 36 s	86 min 14 s	46 min 59 s	39 min 28 s	61 min 35 s	31 min 55 s
8	7 min 24 s	7 min 49 s	7 min 07 s	4 min 23 s	4 min 44 s	4 min 15 s	70 min 36 s	97 min 44 s	55 min 02 s	42 min 51 s	62 min 19 s	30 min 34 s
Mean	7 min 11 s	7 min 45 s	6 min 58 s	4 min 40 s	5 min 15 s	4 min 30 s	57 min 42 s	88 min 03 s	44 min 10 s	36 min 56 s	53 min 59 s	28 min 34 s
with general anesthesia												
^a^9	7 min 36 s	8 min 04 s	7 min 07 s	4 min 34 s	5 min 34 s	4 min 21 s	93 min 09 s	122 min 23 s	81 min 21 s	32 min 52 s	38 min 59 s	28 min 09 s

^a^Beam delivered under general anesthesia. n/a = not applicable, AYA = adolescent and young adult.

All the ipCSI treatments were performed without any prolongation of the radiation treatment period. The mean time from the start to the end of the all beam delivery was 37 min 39 s ± 2 min 24 s (minimum to maximum: 22:49–42:51). The mean time spend in the treatment room was 56 min 46 s ± 2 min 03 s (minimum to maximum: 54:34–60:52) for AYA patients, and 57 min 42 s ± 5 min 37 s (minimum to maximum: 43:24–70:36) for four patients under 15 years of age without general anesthesia, and 93:09 for one patient under 15 years of age with general anesthesia.

No patients experienced gastrointestinal or neurological acute toxicity classified as Grade 3 or more. Grade 3 or higher acute hematological adverse events during the 4-week period after ipCSI were observed in two patients among those under 15 years of age and in one among four AYA patients (Table 1).

**Figure 3.**
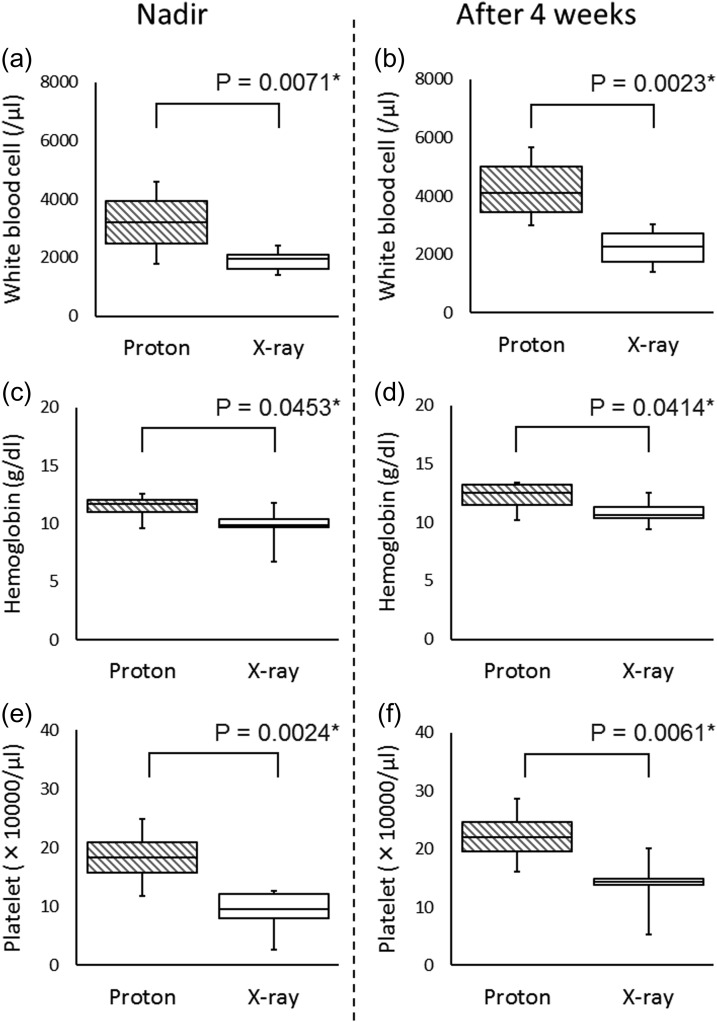
Comparison of acute hematological toxicity between AYA patients who received ipCSI and AYA patients who received photon CSI. (a) The nadir of white blood cell counts during the 4 week period after the end of CSI, (b) the white cell blood counts at 4 weeks after the end of CSI, (c) the nadir of hemoglobin during the 4 week period after the end of CSI, and (d) the hemoglobin at 4 weeks after the end of CSI, (e) the nadir of platelet counts during the 4 week period after the end of CSI, and (f) the platelet counts at 4 weeks after the end of CSI.

In the comparison of patients treated with VBSipCSI vs those treated with photon CSI, the nadir of the WBC count during the 4-week period after the end of CSI was significantly higher with VBSipCSI (3200 ± 607/μl) than photon CSI (1888 ± 120/μl) (*P* = 0.0071, Fig. 3a). The WBC counts 4 weeks after the end of CSI were significantly higher with VBSipCSI (4350 ± 699/μl) than photon CSI (2263 ± 231/μl) (*P* = 0.0023, Fig. 3b). Significant difference was found in the nadir of the hemoglobin during the 4-week period after the end of CSI (11.4 ± 0.6 g/dl, 9.8 ± 0.5 g/dl, *P* = 0.0453, Fig. 3c); the hemoglobin level 4 weeks after the end of CSI was also significantly higher with VBSipCSI than with photon CSI (12.2 ± 0.7 g/dl, 10.8 ± 0.3 g/dl, *P* = 0.0414, Fig. 3d). The nadir of the platelet counts during the 4-week period after the end of CSI was significantly higher in VBSipCSI (183 000 ± 27 000/μl) than in photon CSI (93 000 ± 12 000/μl) (*P* = 0.0024, Fig. 3e). The platelet counts 4 weeks after the end of CSI were significantly higher with VBSipCSI (222 000 ± 26 000/μl) than with photon CSI (139 000 ± 14 000/μl) (*P* = 0.0061, Fig. 3 f).

No apparent correlation between the prescription dose in ipCSI and the grade of toxicity was found.

## DISCUSSION

The incidence of Grade 3 or more acute diarrhea was significantly reduced to 23% in psCSI from 54% in photon CSI (*P* = 0.023) in Song *et al.*’s series [16]. The incidence of Grade 3 or more acute gastrointestinal toxicity was 0% (0/6) in psCSI in MacEwan *et al.*’s series [7]. Our study showed that gastrointestinal toxicity with ipCSI (0/9) was comparable with that found in MacEwan’s study with psCSI.

It has been controversial whether psCSI can reduce hematologic toxicity compared with photon CSI for pediatric patients. Song *et al.* have suggested that Grade 3 or higher grade thrombocytopenia was less common in psCSI (*P* = 0.012); however, WBC counts and hemoglobin levels were not more affected by psCSI than by photon CSI in pediatric patients [16]. McGovern *et al.* reported that 44% (6/14) of pediatric patients with atypical teratoid/rhabdoid tumors experienced Grade 3 or higher-grade hematological toxicity after psCSI [17]. MacEwan *et al.* have shown that 67% (4/6) of infants experienced Grade 3 or more leucopenia using psCSI, even with VBS [7].

Studies are now clearly demonstrating a reduction in hematological toxicity in psCSI with VBS, compared with photon CSI, for AYA patients. Barney *et al.* have reported that the incidence of Grade 3 or more hematological toxicity was as low as 13% (5/40) in AYA patients who received psCSI with VBS [8]. Our results for AYA patients with VBSipCSI (1/4) are similar to those obtained in their study. Brown *et al.* reported that psCSI with VBS reduced hematologic toxicity compared with photon CSI for AYA patients with medulloblastomas [2]. In their study, the median percentage of baseline WBCs, hemoglobin, and platelets at their nadir for patients receiving psCSI with VBS compared favorably with their previous results for photon CSI (WBCs 55% vs 46%, *P* = 0.04; hemoglobin 97% vs 88%, *P* = 0.009; platelets 65% vs 48%, *P* = 0.05). The present study suggested that VBSipCSI was also effective in reducing the severity of myelosuppression compared with photon CSI in AYA patients, in terms of the nadir of the WBC count, hemoglobin, and platelets counts during the 4 weeks following CSI.

However, due to the variety in the co-administered chemotherapy and the differences in the doses delivered to the vertebral bodies, rigorous evaluations of the association between hematological toxicity and doses to the vertebral bodies were difficult to perform. The chemotherapy-scheduling differences between the VBSipCSI and photon CSI may have also influenced the results. These factors need to be evaluated in future studies. It is also important to evaluate the transition of myelosuppression during high-intensity chemotherapy after 36.0 Gy (RBE) VBSipCSI, which is often difficult after photon CSI.

Farace *et al.* have reported that the treatment time from the start to the end of irradiation was 32 min, and the time in the treatment room was 67 min on average in supine CSI in pediatric patients by proton pencil-beam scanning [18]. The mean treatment time and in-room time obtained in our study was comparable with their results for patients who did not receive general anesthesia (treatment time, 37 min and in-room time, 57 min). Questions remain as to whether the dose distributions at the field junction are robust enough when ipCSI is used, because a patient’s conditions can change during the treatment (the treatment time is still >30 min). Our results suggest that it is meaningful to develop a method for reducing the time for set-up of the patients further, because the actual beam-on time is less than one-third of the total treatment time. Recently, Mori *et al.* have shown that their new patient position verification software using 2D–3D image registration is useful in reducing the time for set-up in carbon beam therapy [19]. We think this kind of approach will also be useful for reducing the treatment time and the robustness of the dose distribution for our ipCSI system in the future.

In summary, we confirmed that ipCSI was deliverable in a reasonable time with sufficient HI both for children and AYA patients. Using VBSipCSI, AYA patients experienced a lower incidence of serious acute hematological toxicity than AYA patients treated previously with photon CSI in our institution. The number of patients in this study was too small to draw any definite conclusions; however, our results are encouraging and support further studies on VBSipCSI. More clinical data is required, with longer follow-up periods.
